# Supplementary material for: Cold Atmospheric Plasma Activates Selective Photothermal Therapy of Cancer
Source: Molecules. 2022 Sep 13;27(18):5941. doi: 10.3390/molecules27185941 (PMC9502787; doi:10.3390/molecules27185941)
Supplement: Supplementary file 1 [file molecules-27-05941-s001.zip › molecules-1861085-supplementary.pdf]

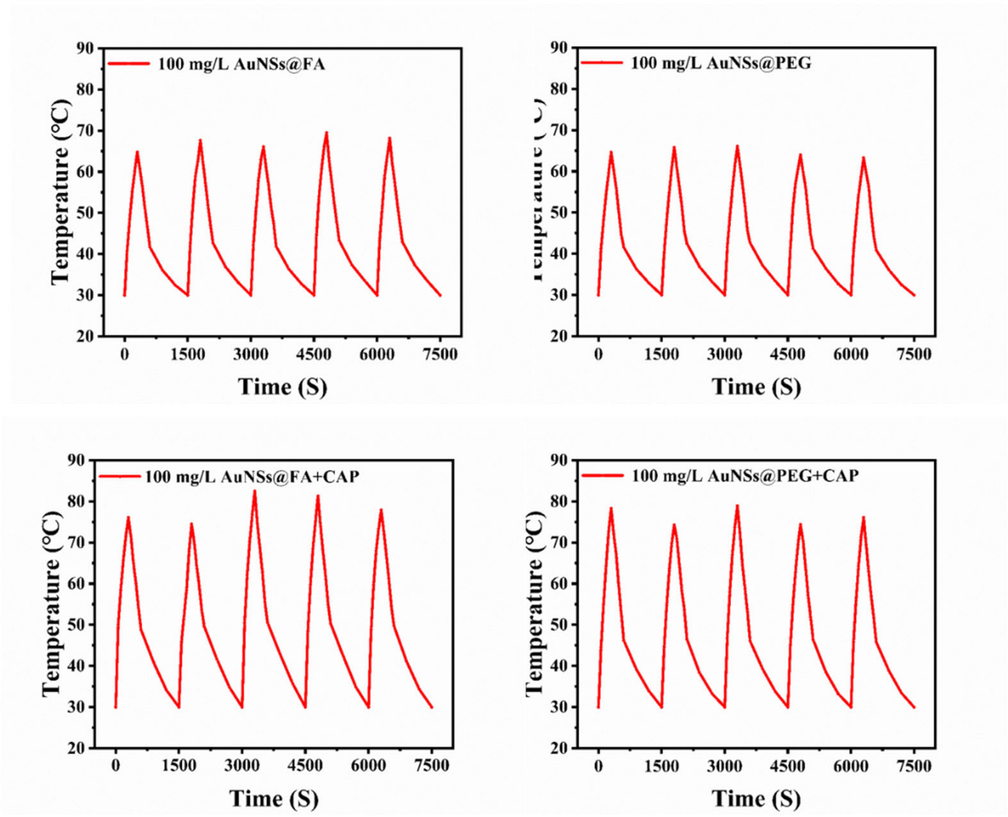

Figure S1: Photothermal stabilities of 100 mg/L AuNSs@FA, AuNSs@PEG, AuNSs@FA+CAP, and AuNSs@PEG+CAP.

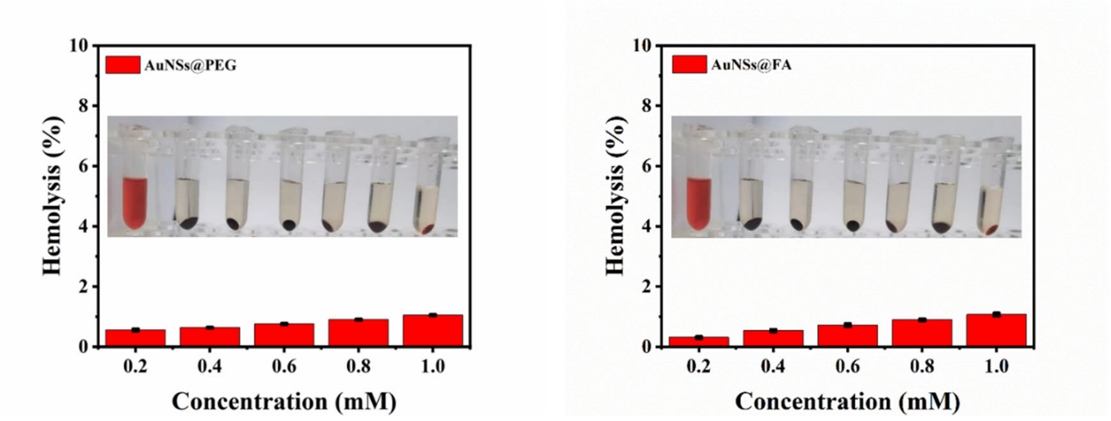

Figure S2: Hemolysis activity in different Au concentrations of AuNSs@PEG and AuNSs@FA.

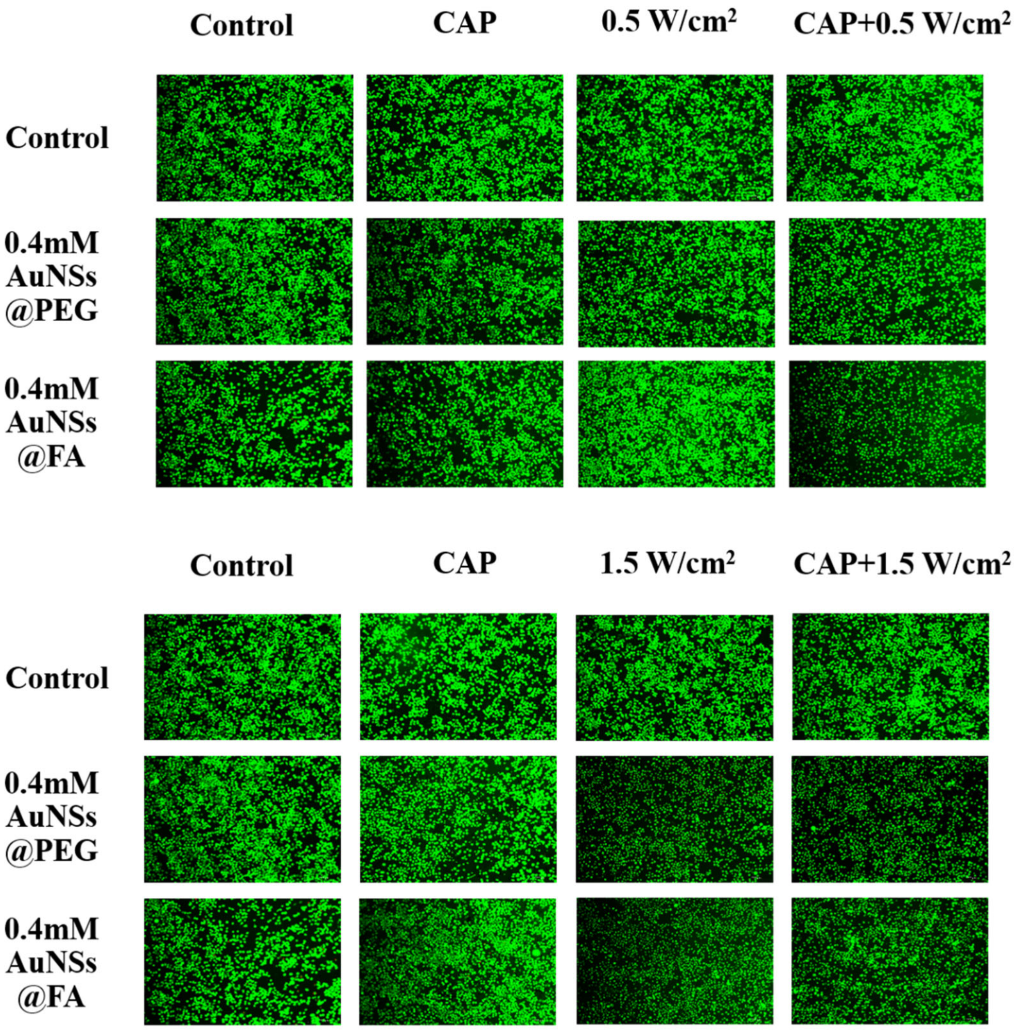

Figure S3. LIVE/DEAD assay of L929 cells after different treatments at an Au concentration of 0.4 mM.

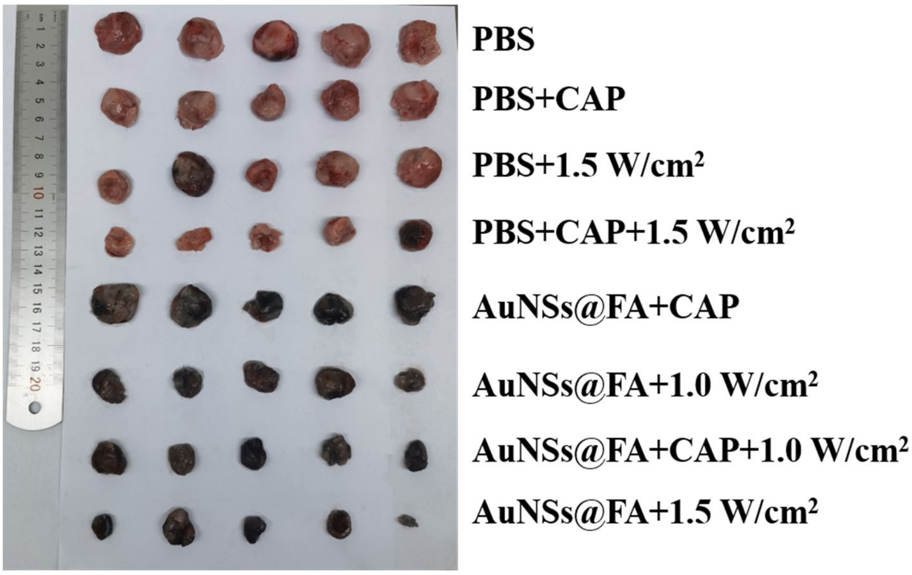

Figure S4. Images of tumors after intraperitoneal injections of PBS and AuNSs@FA with different treatments at day 14.

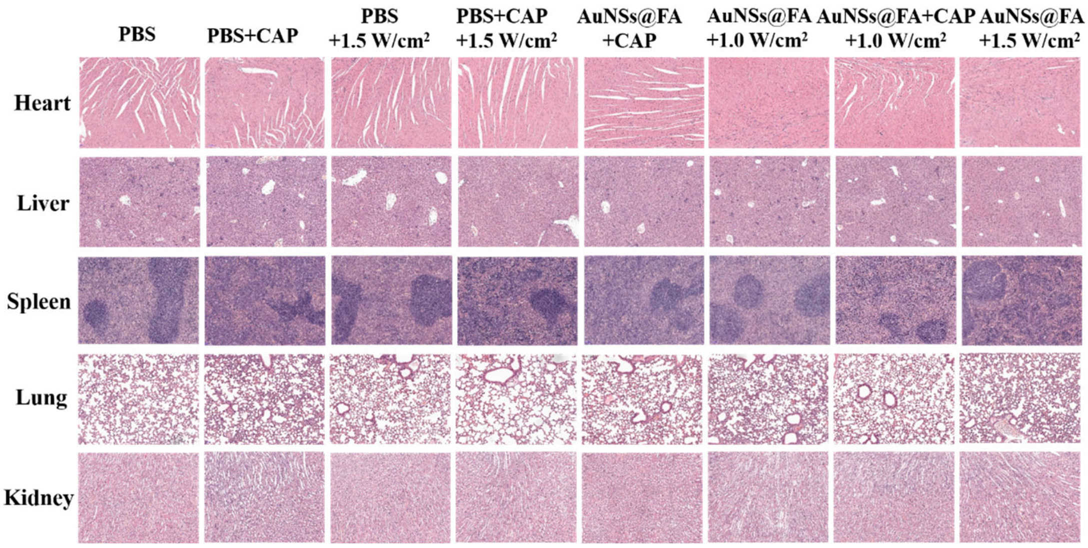

Figure S5. H&E-stained images of major organs of tumor-bearing mice after the various treatments.

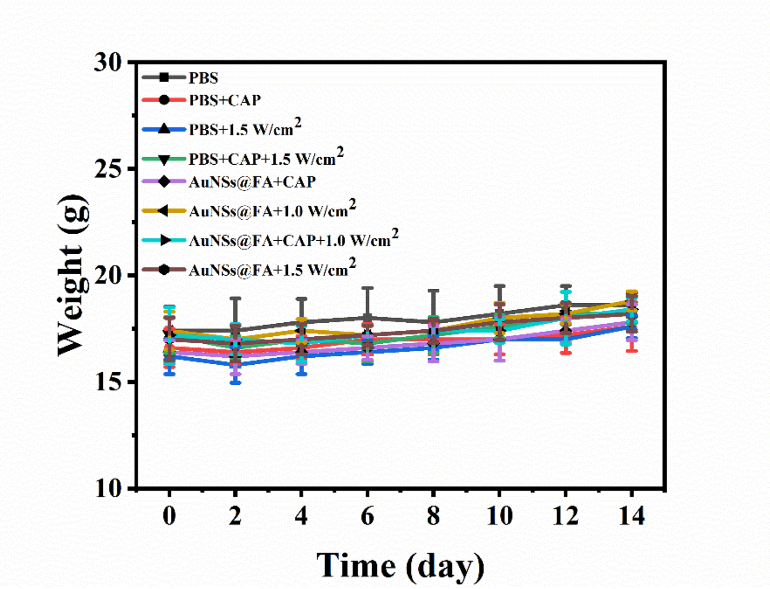

Figure S6. Weights of the tumor-bearing mice during the treatment period.
